# Splenic Artery Infarct Requiring Surgery: A Rare Complication of COVID-19 Infection

**DOI:** 10.1155/2022/3391405

**Published:** 2022-11-30

**Authors:** Ioannis Dimitriou, Nikolaos Christodoulou, Kleanthis Chatzimargaritis, Aristidis Kaikis, Eirini Kasti, Georgios Triantos

**Affiliations:** ^1^1st Surgery Department, General Hospital of Rhodes, Rhodes, Dodecanese, Greece; ^2^Special Unit of Infectious Diseases, General Hospital of Rhodes, Rhodes, Dodecanese, Greece; ^3^Radiological Department, General Hospital of Rhodes, Rhodes, Dodecanese, Greece

## Abstract

*Introduction*. Coronavirus disease (COVID-19) from SARS-CoV-2 infection is linked to a hypercoagulable state, leading to arterial and venous thrombotic events, of which pulmonary embolism is the most frequent. However, arterial thromboembolisms may also occur as visceral infracts in unusual sites, such as the renal, splenic, and intestinal arteries. *Case Report*. A 46-year-old unvaccinated male with a COVID-19 infection was admitted to the COVID-19 isolation ward with symptoms of respiratory infection. He complained of epigastric pain and fever for several days; radiological imaging of the abdomen revealed complete splenic arterial occlusion due to a large infarct. He was treated with low molecular weight heparin (enoxaparin) in therapeutic doses, resulting in minimal improvement. However, the pain worsened, and eventually, a laparotomy and splenectomy were performed. He was hospitalized for another 36 days before he was discharged in good condition. A second surgery was performed to remove a noninfected encapsulated hematoma from the subdiaphragmatic space. The patient remained healthy afterward, with no relapses. *Discussion*. Although rare, the number of cases of visceral infarcts in COVID-19 patients has increased. Splenic artery infarct is an exceptional case of acute abdominal pain that can be treated successfully with anticoagulant medication. Splenectomy may be required to manage refractory pain after failure of conservative management.

## 1. Introduction

Coronavirus disease-2019 (COVID-19) is a viral infection caused by a member of the coronavirus family, the severe acute respiratory syndrome coronavirous-2 (SARS-CoV-2) [1, 2]. The disease was first reported in China in December 2019; since then, it has spread all over the world, turning into a pandemic, causing millions of deaths and infecting more than five hundreds of millions of people [3–5]. The clinical presentation of COVID-19 may range from asymptomatic disease to severe illness, but the virus predominantly attacks the pulmonary system, leading to acute lung injury and diffuse alveolar damage [3]. However, it has been well established that the effects of COVID-19 are not restricted to the lungs and can involve multiple organs, progressing into a systemic disease [1, 3].

SARS-CoV-2 infection has been linked to a hypercoagulable state, leading to diverse arterial and venous thrombotic events [6–8]. The latter has been recorded in several organs, such as venous thromboembolism and disseminated intravascular coagulation (DIC) [6, 9, 10]. Of these events, pulmonary embolism is the most prevalent and may be present, even in the absence of classic respiratory symptoms and fever [8, 11, 12]. On the other hand, arterial thrombotic events are less commonly described [6, 9, 10]. Arterial thromboembolism encompasses a variety of conditions, including myocardial infarction, stroke, and visceral infarcts, such as renal, splenic, and intestinal infarcts [2, 13, 14]. Despite their rarity, abdominal visceral infarcts are increasingly being detected and documented as the disease is spreading. Herein, we present the case of a middle-aged man with COVID-19 disease and severe acute splenic artery occlusion warranting surgery despite receiving therapeutic anticoagulants.

## 2. Case Presentation

**Figure 1:**
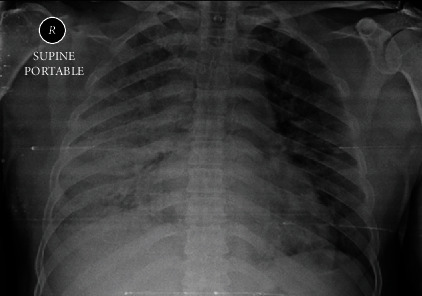
Chest X-ray of the patient from the day he was admitted to the hospital.

A 46-year-old Caucasian male presented to the emergency department of a secondary care hospital complaining of a gradually progressing shortness of breath, fever, dry cough, and dyspnea. He did not receive any vaccination against SARS-COV-2 and had tested positive for COVID-19 one week before his presentation, but he did not seek any medical attention since the appearance of symptoms. On clinical examination, the patient appeared tachypneic, stressed, and had severe hypoxia when breathing air (oxygen saturation (SpO_2_) = 80%, partial pressure of oxygen (pO_2_) = 52.6 mmHg), and febrile (body temperature > 37.5°C). The initial X-ray of the chest revealed bilateral diffuse infiltrations in the middle and lower pneumonic fields, more so on the right lung (Figure 1).

Other laboratory tests were unremarkable (D-dimer and coagulation times were normal), and so were the rest of his vital signs. Although he had a history of chronic asthma, he was not taking any medication and remained free from asthmatic crisis for years. Additionally, he reported a history of chronic hepatitis B (HBV) without having any symptom relevant to this chronic infection. Also, he mentioned having depression-like symptoms without treatment.

**Figure 2:**
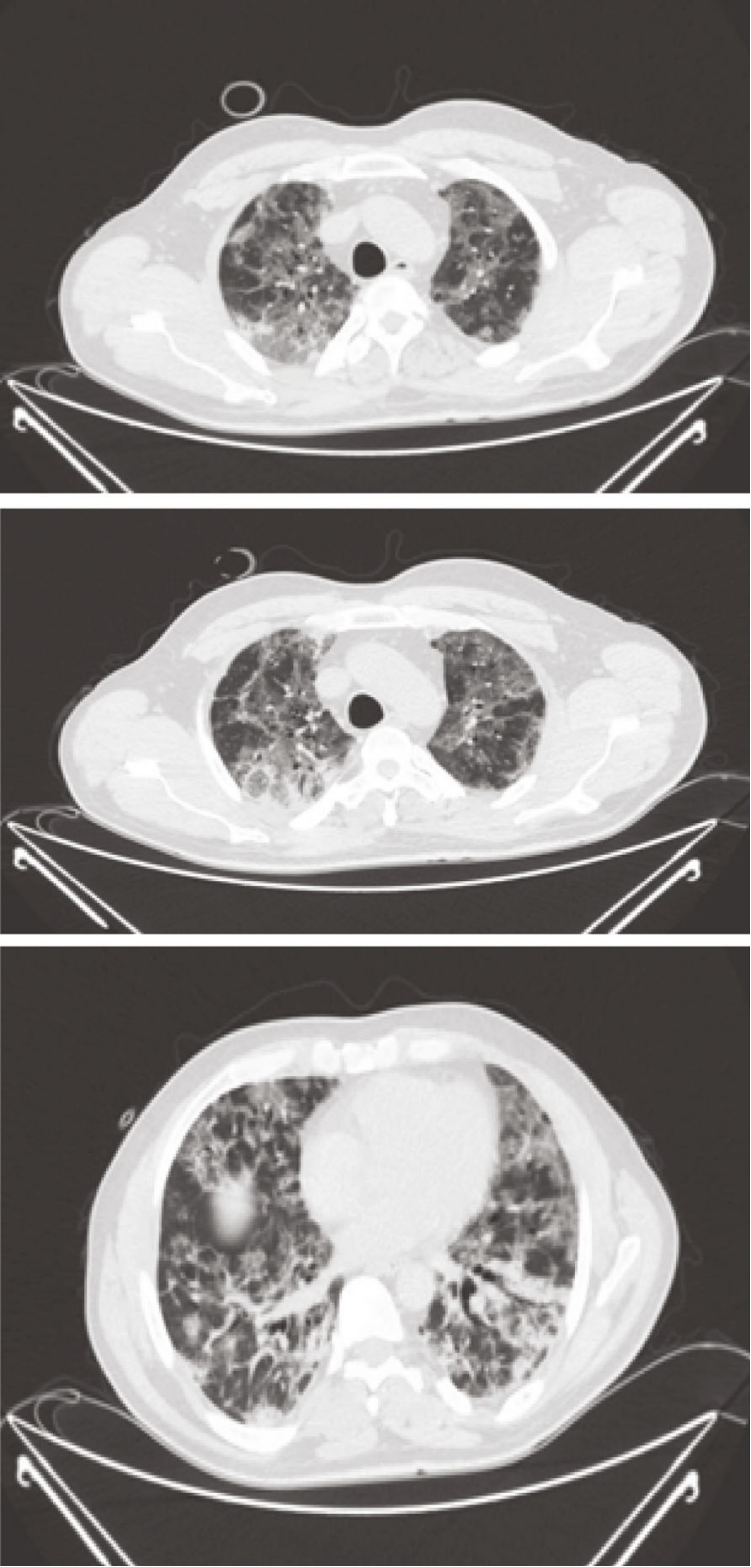
Chest CT shows pathology typical for SARS-COV-2.

The patient was transferred immediately to the special isolation ward made for the coronavirus-infected patients, where he was put on a high flow nasal cannula (HFNC) for high flow oxygenation. He remained on HFNC for 14 days and was then switched to 50% oxygenation with a venturi mask. During the first week, he was febrile, which resolved in the second week. During the hospital stay, he complained of mild epigastric pain, which was initially related to the cough; when the symptoms worsened, he was scheduled for computed tomography (CT) of the abdomen and the thorax (on day 2 of hospital admission). The first CT scan did not show any abdominal pathology, but the pulmonary findings of infiltrations, pyknosis, diffuse ground-glass opacities, and diffuse alveolar damage were typical of a SARS-COV-2 infection (Figure 2).

**Figure 3:**
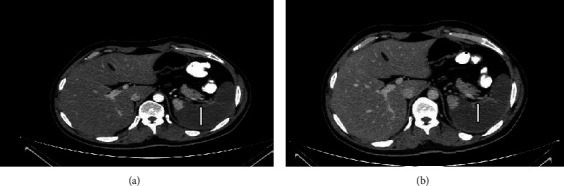
Abdominal CT shows artery occlusion and complete absence of blood circulation on the spleen. White arrow shows the infract after the enhancement of contrast material at the (a) arterial and (b) venous phase.

The patient's epigastric pain was constant and could not be relieved with any drug; eventually, the pain intensity increased so much as to hamper the patient's resting phase. A second CT scan of the abdomen was done on day 14 which revealed a complete obstruction of the splenic artery from a large splenic infract and complete absence of blood circulation in the spleen (Figure 3).

It was decided that the patient be managed conservatively with low molecular weight heparin (LMWH)—enoxaparin 6000 units: 0.6 mL × 2 subcutaneously (SC). He had minimal relief from pain and was admitted for portosplenic triplex and abdominal ultrasonography (US), which revealed that the splenic artery obstruction was complete and there was normal flow of blood inside the splenic and portal vein; the rest of the findings were unremarkable. Treatment with enoxaparin was continued for six days; however, on the seventh day (day 21 of hospital admission), the patient's health deteriorated clinically. Epigastric pain increased, and pulmonary function declined, accompanied by high fever and unilateral pleuritis. A surgical consultation was sought, and emergency surgery was scheduled the same day.

**Figure 4:**
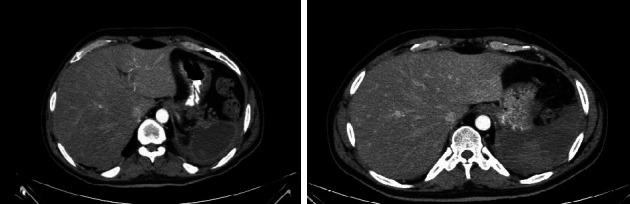
Abdominal CT shows a large collection of liquid on the left subdiaphragmatic space that contains solid material and is encapsulated.

The patient underwent laparotomy and splenectomy, which went well; his postoperative course was uneventful. All postoperative laboratory parameters were within normal ranges besides an isolated elevation in C-reactive protein (CRP) levels (32 mg/dL at the 5^th^ postoperative day (day 26 of admission)). The patient also had a mild fever for a week postoperatively. Eventually, he was fully mobilized without the need for any supplementary oxygen. However, despite the improvement in his clinical condition, the patient continued to be febrile. On the 11^th^ postoperative day (day 32 of admission), he again complained of abdominal pain and was scheduled for a CT scan of the lungs and abdomen, which revealed a large fluid collection in the left subdiaphragmatic space that contained a solid encapsulated lesion with peripheral enhancement using intravenous contrast material and left pleural effusion accompanied by atelectasis of the left lung (Figure 4).

Initially, the symptoms were believed to be due to the contamination of a hematoma in the splenic bed. Blood cultures were taken, and newer antibiotics were prescribed. The patient was followed-up for another 12 days until another surgical consultation was sought. Throughout this period, he experienced waves of febrile and afebrile episodes; abdominal pain, especially in the left subcostal area, was steady but well tolerated. All blood cultures tested negative for any possible microorganisms. All vaccines recommended after splenectomy against pneumococcus, *Haemophilus influenzae*, and meningococcus B and C were administered on the appropriate postoperative day according to the national vaccination policy of our country [15]. The attending consultant from the infection pathology unit, in coordination with the surgical team, decided to proceed with a second operation to evacuate the collection, which seemed to be the underlying cause of fever and pain.

The second operation was undertaken 24 days after the first surgery (i.e., on day 45 of admission). The incision scar from the previous surgery was used to approach the peritoneal cavity; a well-encapsulated hematoma was evacuated and copiously washed out until clean water could be suctioned from the suction device. There was no pus or dead tissues in any of the peritoneal spaces. Culture samples were taken from the extracted specimen, and a silicone drain was placed at the site of the splenectomy.

**Figure 5:**
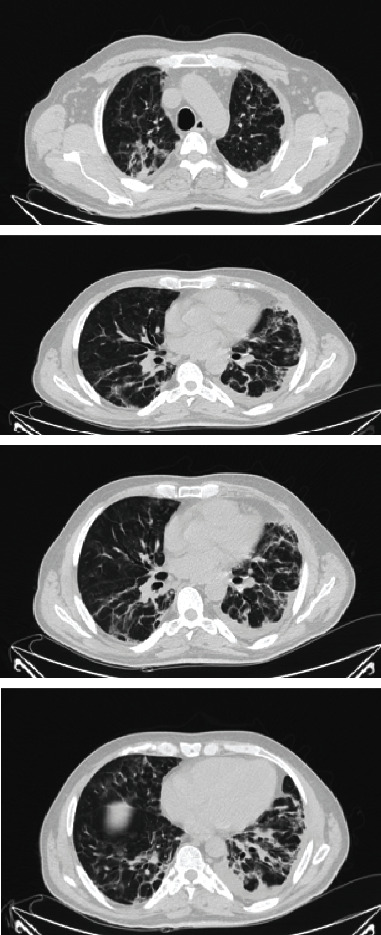
Chest CT showing permanent damage to the lungs by the coronavirus.

The postoperative course was uneventful, and the patient remained afebrile for 10 days before being discharged from the hospital on the 56^th^ day of his admission. Three days before his discharge, another CT scan of the chest was done, which confirmed permanent damage to his lungs caused by the coronavirus, a small residual hematoma in the left upper abdomen, and two small encapsulated collections at the base of the left lung (Figure 5).

At the one-month follow-up visit, the patient was healthy, with no pulmonary difficulties or any other symptoms. He complied with all postoperative instructions regarding the postsplenectomy vaccination program, as well as the antithrombotic (enoxaparin 0, 6 IU for 6 months) and antibiotic (amoxicillin 500 mg for 2 years) treatments; he was eventually vaccinated for coronavirus. It was also suggested to him to have blood tests for platelets, liver function, and clotting times every 3 months for the first year postoperatively and then consult a hematologist for further instructions. Seven months after his discharge, he is doing well, with no further complications (Table 1).

## 3. Discussion

Splenic infarction occurs as a result of obstruction of the splenic artery or its branches due to thrombosis or embolism [7, 8]. The most common causes of arterial obstruction are neoplasms, cardioembolic events, and hematological diseases; however, this event has also been linked to acute viral infections, which often trigger states of hypercoagulability [16–19]. SARS-CoV-2 infection is also associated with a prothrombotic state and an increased risk of venous and arterial thromboembolism [8, 11, 20].

In the case of a SARS-CoV-2 infection, endothelial integrity and normal venous blood flow may be dysregulated due to endothelial or vascular injury, resulting in venous stasis [8, 21–23]. Additionally, the cytokine storm that accompanies the response to coronavirus infection may activate the coagulation system, causing systemic inflammation, endothelial cell activation, and the release of tissue factors, predisposing the tissue to thrombosis [2, 6, 17, 20, 22]. Also, there is an abnormal increase in the levels of coagulation parameters, such as D-dimers, fibrin degradation products (FDP), thromboelastographic (TEG) values, and prolonged coagulation times (like activated partial-thromboplastin time, aPTT), which may be associated with coronavirus infection [11, 16, 24–27]. Platelet function is also known to be affected by COVID-19 disease, i.e., abnormal platelet function may explain arterial thrombosis resulting from an increase in platelet activation at suboptimal thresholds and an increase in platelet aggregation in COVID-19 patients [20, 25]. Despite these hypotheses, the exact mechanism underlying coronavirus-induced coagulopathy remains undetermined [16, 28, 29].

The most common thrombotic event reportedly associated with COVID-19 is pulmonary embolism [11, 12, 16], whereas splanchnic vein or artery thrombosis is less common, and splenic artery infarct and occlusion are rarely reported [7, 13, 16]. Splenic infarction is an unusual cause of abdominal pain and is mostly caused by an underlying hematological condition [8, 16]. Other causes of splenic infarction are cardiovascular disorders, coagulation disorders, hypertension, diabetes mellitus, and viral infections [13, 16]. Splenic artery infarct often manifests with medium to severe abdominal pain and tenderness in the left hypochondrium or left upper abdominal quadrant, fever, nausea, vomiting, besides occasional dyspnea, tachypnea, and tachycardia [2, 13]. The imaging modality of choice for diagnosis is a contrast-enhanced abdominal CT scan [8, 17, 19, 30].

LMWH is the first-line treatment for vesical artery thrombosis, and the same treatment has been followed in most cases of splenic artery thrombosis reported in the literature [31, 32]. Other treatment therapies include the use of antiplatelet agents (acetylsalicylic acid, ASA, and clopidogrel) and recently invented anticoagulant agents that trigger thrombin (apixaban and dabigatran). All these medical agents can be used for both prophylaxis and treatment depending on the patient's features and medical condition. However, failure of medical treatment in splenic artery occlusion is a strong indication for surgery, besides other scenarios such as hemorrhage, aneurysm, or a splenic abscess due to COVID-19 infection [28, 32, 33]. In our patient, we administered LMWH therapy in therapeutic doses for seven days until the patient's clinical deterioration forced us to proceed to laparotomy and splenectomy. In situations where the spleen has to be removed abruptly, the risk of postsplenectomy infection and sepsis is higher [2, 34].

We also conducted a literature search across the Medline, PubMed, and Google Scholar databases regarding splenic artery infarct or occlusion in COVID-19 patients and found that such an infarct due to thrombosis appears seldom, with only 45 cases reported so far. Of these cases, 30 were male patients and 12 were female, while the patient's gender was not mentioned for three cases (Table 2) [2–14, 16, 19–32, 34–44].

We found that the treatment and prevention strategies for such thrombotic events in the presence of COVID-19 infection were debatable [11, 31]. The standard of care is antithrombotic treatment using medicines such as heparin, LMWH, ASA, apixaban, rivaroxaban, and antiplatelet agents such as clopidogrel and ticagrelor [20, 45]. However, controversy arises as to whether hospital patients with COVID-19 infection without signs of thromboembolism should receive prophylactic or therapeutic anticoagulation [11, 24]. In our literature search, we found that 38 of the 45 cases received medical treatment—eight received heparin, 29 received LMWH (mainly enoxaparin), and in the remaining 16 cases, other anticoagulation medicines (ASA, clopidogrel, etc.) were used. Two of the patients underwent conservative treatment with no further details, while the treatment followed for another two patients was not reported. In only 3 cases, which were reported by Al-Ozaibi et al. [34], Besutti et al. [35], and Imam and Hammond [36] did the clinician proceed to splenectomy for treating a severe splenic infarct. Our patient is only the fourth case that required a splenectomy as an essential component of treatment. Furthermore, we discovered that in 28 of the 45 reported cases, there were simultaneous thrombosis and infarcts in other tissues, such as the kidneys, brain, aorta, lungs, bowel, and splenic vein. In 14 cases, the patients had isolated thrombosis in the splenic artery, and in 3 cases, there was no record of any simultaneous thrombosis. Fortunately, the clinical outcome of the splenic infarct was favorable in most cases, with 31 of 45 cases reporting improvement or full recovery of the splenic artery occlusion. Unfortunately, five patients were diseased due to sepsis or heavy medical comorbidities. In nine patients, the clinical outcomes were not reported.

To the best of our knowledge, this case report is only the second case in Greece of a patient developing a splenic artery infarct due to COVID-19 infection and only the 4^th^ case so far that warranted surgery despite conservative treatment with anticoagulant agents, such as LMWH. Based on the existing literature, we can conclude that thrombosis from COVID-19 infection is not unusual and can occur regardless of the use of antithrombotic agents [27, 37, 38]. Moreover, an increase in D-dimer levels is not sufficient to diagnose arterial or venous thrombosis owing to the considerable inflammatory reaction and significant rise in D-dimer levels following COVID-19 infection [2, 16]. Nevertheless, the chances of hypercoagulability that accompanies this disease should raise suspicion of thrombotic events in unusual sites, and treatment with anticoagulants should start immediately after diagnosis [14, 24]. A combination of more than two agents is sometimes necessary, and prolonged treatment, even after improvement, is, at times, inevitable [45]. Routine use of LMWH should be followed in patients with SARS-CoV-2 infection, although some authors consider this controversial [11, 27, 31, 38, 39]. There is no consensus regarding the best therapy for arterial occlusion, and different authors have used a variety of medications for treating arterial infarcts after coronavirus infection [21, 28]. However, it is crucial to determine the best therapy for acute arterial thrombosis in which not only the appropriate anticoagulant treatment regimen but also the correct dose is essential to improve prognosis, relieve symptoms, and significantly reduce mortality and disability [21, 40]. In addition, splenectomy is a risk factor for overwhelming bacterial sepsis. If this situation exposes patients with COVID-19 infection in greater risk than patients without COVID-19 infection is not known as data are absent in current literature. In advance there is no evidence that absence of spleen places patients in greater risk for COVID-19 infection [46]. Furthermore, from a brief review of the literature, we observed that antithrombotic treatment with LMWH, ASA, and other anticoagulants results in improvement in splenic artery infarcts, causes recanalization of the splenic artery, and that splenectomy is required only in the case of clinical deterioration of the patient [20, 30, 31, 33, 41, 47]. While we await further data from clinical trials regarding treatment guidelines, a reasonable approach would be to continue with regular-dose thromboprophylaxis in all COVID-19 patients and to provide therapeutic anticoagulation in patients with confirmed thrombotic events [2, 14, 21, 24, 35, 39].

## 4. Conclusion

Emergency physicians, clinicians, and general surgeons must be very cautious of the thrombotic events associated with SARS-CoV-2 occurring in unusual sites, such as the splenic artery. Splenic artery thrombosis or infarction may be suspected in COVID-19 patients who suffer from acute upper abdominal pain, especially in the epigastrium and the left hypochondrium, regardless of the absence of prothrombotic states or hematological abnormalities. Conservative treatment with LMWH is the first-line treatment followed by anticoagulants and antiplatelet agents. Splenectomy is the last strategy when conservative methods have failed. High index of suspicion is mandatory when COVID-19 infection is accompanied with acute abdominal pain.

## Figures and Tables

**Table 1:** Timeline of clinical sequelae of the patient.

Time	Event
Day 0 (hospital admission)	SARS-CoV-2 infection—fever, dry cough, dyspnea-HNPC
Day 2	Increased abdominal pain—first CT scan (no pathology)
Day 14	Increased abdominal pain—second CT scan (splenic artery infarct and hypoperfusion on the spleen). Gradual respiratory improvement-venturi mask 50%. Start of LMWH (enoxaparin)
Day 17	Portosplenic vein triplex: incomplete splenic artery obstruction, normal blood flow in splenic and portal vein
Day 21	Severe abdominal pain—first surgical operation: laparotomy and splenectomy
Day 26	Fever and high CRP
Day 28	No need for supplementary oxygen
Day 32	Fever—new onset of abdominal pain Third CT scan (hematoma at the splenic bed)
Day 45	Second surgical operation: evacuation of the hematoma, washing of the peritoneal cavities and drains
Day 53	Revision CT scan of the chest: confirms the damage to the lungs that was made by coronavirus
Day 56	Discharge from hospital

**Table 2:** Literature review of cases of COVID-19 with splenic infarcts.

Author	Sex	Age	Clinical presentation	Treatment	Outcome
Atici and Akpinar [2]	M	45	Splenic infarct	LMWH (enoxaparin) followed by ASA and ticagrelor	Recovered
Dennison et al. [3]	M	70	Splenic infarct and bilateral rectus sheath hematoma	LMWH (complication)	Improvement
Dagistanli and Sonmez [4]	F	42	Splenic infarct secondary to splenic vein thrombosis and splenic abscess	LMWH (enoxaparin) percutaneous drainage and suction	Improvement
Redekar et al. [5]	M	55	Multiple splenic infracts	UFH LMWH plus acenocoumarol	Improvement
Karki et al. [6]	M	32	Splenic infarction	Supportive care	Splenic rupture-ICU admission—outcome not reported
Moradi et al. [7]	F	59	Splenic infarct and limb ischemia (foot)	Heparin followed by rivaroxaban, ASA, and clopidogrel	Improvement
Al-Mashdali et al. [8]	M	43	Splenic and renal infarct (AKI)	Heparin and warfarin	Improvement
Tranca et al. [9]	F	30	Postpartum	LMWH plus antiplatelet agents	Improvement
Singh P. and Singh S. [10]	M	40	Aortic thrombus and splenic infarct	LMWH followed by rivaroxaban	Improvement
Agha et al. [11]	M	60	Splenic infarction	Heparin for 24 h IV infusion and then enoxaparin SC. Rivoroxaban on discharge	Recovered
Pistor et al. [12]	M	17	Splenic artery thrombosis and acute stroke	LMWH (enoxaparin) followed by aspirin	Improvement
Prentice et al. [13]	M	50	Splenic infarct	UFH (heparin) followed by enoxaparin	Improvement
Yildiz et al. [14]	M	68	Splenic infracts plus pneumonic emboli	LMWH	Improvement
Castro et al. [16]	M	67	Splenic infarct	LMWH (enoxaparin) followed by rivaroxaban	Improvement
Sztajnbok et al. [19]	F	60	Splenic and aortic thrombosis	LMWH (enoxaparin) followed by warfarin	Improvement
Gold et al. [20]	M	59	Extensive thrombosis of the thoracic and abdominal aorta, celiac trunk, hepatic artery, splenic artery and vein, spleen, and right kidney infarctions	LMWH (therapeutic dose)	Deceased from sepsis 1 month after event
Gold et al. [20]	M	70	Multiple brain infarctions, vascular occlusions of both lungs, spleen, and kidney infarctions	LMWH (therapeutic dose)	Deceased 2 weeks after event
Gold et al. [20]	M	78	Multiple pulmonary emboli with lung infarctions, spleen, and bilateral kidney infarctions	LMWH (therapeutic dose)	Deceased 3 days after event
Mavraganis et al. [21]	M	64	Simultaneous infracts at the splenic vein, artery, renal artery, and aortic thrombi	LMWH plus acetylsalicylic acid	Recovered
Vidali et al. [22]	F	70	Splenic artery occlusion and splenoportal-mesenteric axis thrombosis	LMWH	Not reported
Roquetaillade et al. [23]	—	—	Not mentioned	Anticoagulation medical therapy	Not reported
Hossri et al. [24]	F	29	Splenic infarct and ischemic stroke	Heparin in continuous infusion	Not reported
Pessoa et al. [25]	M	67	Splenic infarct and ischemic stroke plus pulmonary	No information	Not reported
Pessoa et al. [25]	F	53	Splenic infarction	Not reported	Not reported
Ghalib et al. [26]	F	67	Splenic infarct	UFH (heparin) followed by LMWH	Improvement
Al Suwaidi et al. [27]	F	23	Splenic infarct	Rivaroxaban	Improvement
Norton and Sheikh [28]	M	30	Occlusive thrombus in splenic artery	LMWH (tinzaparine) and warfarin	Improvement
Mathew et al. [29]	M	34	Splenic infarct	LMWH	Improvement
Javaid et al. [30]	M	44	Celiac trunk thrombosis extended to spleen	Supportively (not reported)	Improvement
Ramanathan et al. [31]	M	54	Bilateral renal and splenic infarcts	Apixaban	Recovered
Mahmood et al. [32]	F	27	Postpartum multiple splenic infarcts	LMWH followed by rivaroxaban	Improvement
Al-Ozaibi et al. [34]	M	57	Splenic abscess with rupture	Splenectomy	Died due to sepsis, MOF, and PE
Besutti et al. [35]	M	53	Splenic infarction and left kidney infarction	LMWH	Recovered
Besutti et al. [35]	M	72	Massive splenic infarction and small bowel ischemia	Splenectomy and small bowel resection	Recovered
Imam and Hammond [36]	F	58	Splenic vein, artery, and portal system thrombosis	Percutaneous drainage of the abscess and splenectomy	Not reported
Guven [37]	M	54	Splenic and abdominal aortic thrombosis	LMWH (enoxaparin)	Improvement (complete resolution of thrombus)
Jahromi et al. [38]	M	76	Renal, splenic, and myocardial infarction	Heparin followed by oral anticoagulants	Improvement
Do et al. [39]	M	66	Splenic infarct and infrarenal thrombus	Enoxaparin followed by heparin and enoxaparin	Improvement
Imoto et al. [40]	M	64	Multiple cerebral infracts, bilateral renal infracts, splenic infarction	LMWH	Died on hospitalization
Cetiner et al. [41]	M	71	Splenic infarct and stroke	LMWH (enoxaparin)	Improvement
Fernandes et al. [42]	M	44	Splenic and arterial thrombi	LMWH (enoxaparin)	Improvement
Arslan [43]	F	42	Splenic venous and arterial thrombosis and celiac artery thrombi	Heparin followed by enoxaparin and ASA	Improvement
Rigual et al. [44]	M	53	Splenic, cerebral, and bilateral renal infarcts	LMWH (enoxaparin) followed by acetylsalicylic	Improvement
